# Patterns of Internet Use, and Associations with Loneliness, amongst Middle-Aged and Older Adults during the COVID-19 Pandemic

**DOI:** 10.3390/healthcare10071179

**Published:** 2022-06-23

**Authors:** Anna-Stiina Wallinheimo, Simon L. Evans

**Affiliations:** Faculty of Health and Medical Sciences, School of Psychology, University of Surrey, Guildford, Surrey GU2 7XH, UK; anna-stiina.wallinheimo@surrey.ac.uk

**Keywords:** middle-aged and older adults, coronavirus, internet use, loneliness, digital divide, gender

## Abstract

Loneliness among older adults is a major societal problem with consequences for health and wellbeing; this has been exacerbated by the coronavirus pandemic. The present study investigated associations between internet use, including frequency and type of use, and loneliness in a large UK sample of middle-aged and older adults, aged 55–75 (*n* = 3500) from the English Longitudinal Study of Ageing (ELSA) cohort study. Our findings indicated a clear relationship between the frequency of internet use and subjective loneliness. Those who used the internet more than once a day reported feeling less lonely than those who used the internet once a week or less. We also found that those who used the internet for e-mail communication were less lonely. However, individuals indicated higher levels of loneliness when the internet was used for information searches about health. Regarding sociodemographic factors underlying internet usage, less frequent use was seen amongst individuals who lived alone, people who were not employed, who had lower education levels, and lower sociodemographic status. Additionally, gender differences were found in the type of internet use: males report using the internet for e-mail communication more than females, while females’ internet use for health-related information searches was higher than in males. In sum, findings suggest that intervention strategies that promote internet access amongst middle-aged and older people could be useful for tackling loneliness and point to the groups within society that should be the focus of such interventions.

## 1. Introduction

The COVID-19 pandemic has had significant psychological effects on people’s health and well-being globally. To restrict the spread of the Coronavirus, social distancing and quarantine strategies were employed. In the UK, a nationwide ‘lockdown’ was imposed on 23 March 2020 to cease all unnecessary social contact [1]; the imposition of similar measures around the world has been linked to increased levels of social isolation and loneliness [1,2,3,4]. Loneliness is a situation where the lack of interpersonal relationships is felt as unacceptable or unpleasant [5]. Loneliness has been a widespread consequence of the pandemic-related social restrictions, and this has contributed to the recent rise in mental illness symptomatology seen in adults across the age range [2]. Amongst older adults, feeling socially isolated and lonely raises the risk of depression, fatigue, and sleep disturbance [6,7,8,9]. There is consistent evidence of increased depression and loneliness levels amongst older adults as a result of COVID-19 lockdowns [10]; social isolation has been linked to psychological distress and mental health issues during the pandemic in adults aged ≥ 55 years [11,12]. However, to date, limited work in this age group has been conducted. Studies investigating the mental health impact of lockdowns have tended to focus more on younger age ranges or recruit participants from across the adult age range. Insights specific to middle-aged and older adults are lacking; although, a recent longitudinal study showed increased depression levels in UK adults aged over 50, showing that depression levels increased while lockdown measures were in place compared to pre-pandemic, with females, those living alone, and those with a long-standing illness at highest risk [13].

A potentially important factor concerns technology use, which can help in maintaining connectedness and alleviating loneliness amongst older age groups. The internet and digital technology have transformed society, becoming integral to public, economic, and social life and the way we communicate, learn, entertain, and access information and services. The COVID-19 pandemic has accelerated this trend, as social restrictions have led to an even greater reliance on online services. However, internet access and use are uneven across the population, and many people remain digitally excluded. These people tend to be older and less educated and are more likely to be unemployed, disabled, and socially isolated [14]. Older people have consistently made up the largest proportion of internet non-users [15]. However, internet use could potentially counter the effects of pandemic-related social restrictions on loneliness outlined above, helping to protect the mental health of middle-aged and older people. Pre-pandemic, several cross-sectional studies have linked later life internet use to reduced loneliness [16]. A large-scale cross-sectional analysis of over 60,000 individuals aged 50+ across Europe found a significant association between internet use and decreased loneliness [17]. More frequent internet use has been associated with reduced depression levels amongst older adults both cross-sectionally [18] and longitudinally [19]. Another study linked frequent internet use in later life to better physical and mental health and higher well-being. These links were mediated by reduced loneliness [20]; however, this was based on a sample of only 591. Small sample sizes are an issue in the literature; moreover, most work to date has been limited by only including yes/no measures of use rather than more fine-grained data. Further, there is relatively little data on the effects of different *types* of internet use. This is important: pre-pandemic studies suggest that internet use for communication purposes associates with lower depression and better life satisfaction while using the internet for information access links to higher levels of loneliness [21]. Wallinheimo and Evans (2021) found that more frequent internet use during the COVID-19 pandemic was associated with enhanced quality of life and lower depression scores in adults aged over 55. Importantly, those who reported using the internet for communication purposes (e.g., sending and receiving e-mails) had higher quality of life scores, but use for health-related or government services information search was, in contrast, associated with higher depression symptoms [22].

When looking at internet use and gender, there are currently mixed findings in relation to frequency and type of internet use. Simonova et al. (2020) found that gender was not an important factor in relation to the internet usage habits of older people [23]. However, some previous findings have highlighted that men use the internet more than women [24,25,26]. When it comes to the type of use, there is evidence to suggest that girls’ use of social networking is higher than boys’ [26] and that women’s use for searching for health-related information is higher [27]. It is important to note, though, that most of these studies have focused on the general population or children rather than older internet users. Hence, the current study will appraise gender effects on the type of internet use.

The present study aims to characterize the relationships between internet use (both frequency and type of use) and self-reported loneliness during the pandemic amongst individuals aged 55–75. We used data drawn from The English Longitudinal Study of Ageing (ELSA), a well-established longitudinal cohort study comprising a representative sample of adults aged over 50 living in England [28]. ELSA conducted a COVID-19 sub-study in June/July 2020, where data on various lifestyle and health measures were gathered to investigate the effects of the COVID-19 outbreak. The first objective of the current study was to investigate the role of internet use (both in frequency and type of internet use) in relation to loneliness. Based on previous findings, we predicted associations between more frequent internet use and lower levels of loneliness. Regarding the purpose of internet use, as suggested by prior evidence, we hypothesized that using the internet for e-mail communication would associate with lower loneliness levels while, conversely, using the internet to search for health-related information would associate with increased loneliness. As a second objective, we wanted to further investigate the role of gender in the type of internet use. Finally, the third objective was to ascertain the demographic factors which influence internet use habits in this age range, to provide context to the findings, and to ascertain which social groups are most likely to be ‘digitally excluded’, thus informing potential targeted intervention strategies to increase internet participation amongst older individuals, allowing wider access to the benefits which such participation might offer.

## 2. Materials and Methods

### 2.1. Data and Sample

We used data from The English Longitudinal Study of Ageing (ELSA), a representative cohort comprising individuals aged 50 and over living in England [28]. Data are publicly available at https://beta.ukdataservice.ac.uk/datacatalogue/series/series?id=200011 (accessed on 1 January 2021). In June/July 2020, ELSA conducted a COVID-19 sub-study to investigate the effects of the COVID-19 pandemic on older people in England. Data from this sub-study was used to address the current study aims. The data were accessed under project number 206540 in January 2021.

ELSA participants were contacted by post to take part in a survey lasting about 30 min. A combination of internet and telephone assessments was used. There was a financial incentive to participate (GBP 10). Ethical approval was granted by the Multicenter Research and Ethics Committee (MREC 01/2/91), and ELSA was conducted in accordance with the Declaration of Helsinki; participants gave informed consent to take part. We used the following inclusion criteria: aged 55–75 and currently living in a private household (i.e., not hospitalized or in care), to minimize potential confounds related to age-related health issues. All data were drawn from the COVID-19 sub-study (June–July 2020), apart from the education and wealth quintile, which were drawn from the ELSA wave 9 (June 2018–May 2019). We only included participants with data on all the variables under study here. This meant that we excluded 1149 participants due to missing data (e.g., wealth quintile), yielding a final sample of 3500.

### 2.2. Frequency and Purpose of Internet Use

We used self-reported data regarding the frequency and purpose of internet use. In relation to the frequency of internet use, participants were asked “Since the coronavirus outbreak, on average, how often did you use the Internet or e-mail?” There were six internet use frequency options which we collapsed to form four: (1) more than once a day, (2) once a day, (3) once a week, and (4) less than once a week. We have adopted a similar approach previously using these four categories [22]. Regarding the purpose of internet use, the question asked was “For which of the following activities did you use the Internet in the last 3 months?” Only the participants who used the internet more than once a month were asked: there were ten options given and participants responded yes or no to each, thus it was possible to indicate more than one option: (1) Sending/receiving e-mails; (2) Making video calls or voice calls (e.g., using applications such as Skype, WhatsApp, or FaceTime); (3) Finding information on health-related issues; (4) Managing my finances (online banking, paying bills, and paying taxes); (5) Shopping/buying goods or services; (6) Using social networking sites (Facebook, Twitter, LinkedIn, Instagram, blogging, or Flickr); (7) Reading news/newspaper/blog websites; (8) Streaming TV/videos/radio (BBC iPlayer, Netflix, Amazon Prime, YouTube), listening to music (Spotify, Apple Music), playing online games, or reading eBooks; (9) Getting information about government services (benefits, taxes, a driving license, or passport, etc.); and (10) None of the above.

### 2.3. Subjective Loneliness

To measure loneliness, participants were asked “How often do you feel lonely?”. The possible responses were: (1) never/hardly, (2) some of the time, and (3) often.

### 2.4. Sociodemographic Factors

We included the following predictors in our regression model: living alone or not alone, age, gender, EIMD 2015 score (The Index of Multiple Deprivation 2015 is the official measure of relative deprivation for small areas and neighborhoods in England coded as 1–5 (1 = most deprived), urban/rural living, current employment situation (employed, unemployed, retired, and other), highest education (degree level, higher education, secondary education, and below secondary education), ethnicity as BAME (Black, Asian, and minority ethnic) or non-BAME, and wealth quintile (total net non-pension household wealth, by quintile).

### 2.5. Data Analysis Plan

To investigate the effects of frequency of internet use (more than once a day, every day, once a week, or less than once a week) on subjective loneliness, a between-groups analysis of covariance (ANCOVA) was conducted; all the sociodemographic factors were included as covariates. Then, we conducted a multiple regression to determine the sociodemographic factors affecting the frequency of internet use. To investigate which types of internet use affected subjective loneliness, we conducted a separate multiple regression with all nine types of internet use, and the sociodemographic factors, as predictors. Additional analyses (ANOVAS) were conducted to investigate the role of gender on the type of internet use. Analyses involving the type of internet use were only conducted in the participants who used the internet more than once a month since less-frequent users were not asked those questions.

## 3. Results

### 3.1. Descriptive Statistics

The data comprised of 3500 participants (age: *M* = 67.20, *SD* = 5.54). Females represented 57% of the sample. Table 1 summarizes the characteristics of the whole sample. A total of 6% of the participants ‘often’ felt lonely, 23% were lonely ‘some of the time’, and 71% were ‘hardly ever or never lonely’. In total, 57% reported using the internet more than once a day, 24% used it every day, 7% once a week, and 12% used the internet less than once a week. Amongst the participants who used the internet more than once a month, e-mail communication was the most popular type of internet use (80%), followed by shopping/buying goods or services (68%), making video or voice calls (58%), and managing finances (56%). A total of 52% reported using the internet for reading news, 46% for using social networking sites, 45% for streaming TV, video, or radio, 40% for finding information on health-related issues, and 36% for getting information about government services.

### 3.2. The Effects of Frequency of Internet Use on Subjective Loneliness

A one-way between-groups analysis of covariance (ANCOVA) was conducted with the frequency of internet use (more than once a day, every day, once a week, or less than once a week) as the independent variable and subjective loneliness as the dependent variable whilst controlling for living alone or not alone, age, gender, EIMD 2015 score, urban/rural living, current employment situation, highest education, ethnicity, and wealth quintile. There was a significant main effect of frequency of internet use on loneliness, *F*(3, 3476) = 5.73, *p* < 0.001. A higher frequency of internet use was associated with lower subjective loneliness scores: ‘more than once a day’ (*M* = 1.32; *SE* = 0.01), ‘every day’ (*M* = 1.35; *SE* = 0.02), ‘once a week’ (*M* = 1.43; *SE* = 0.04), and ‘less than once a week’ (*M* = 1.44; *SE* = 0.03). Pairwise comparisons (with Bonferroni correction) identified a significant difference between Group 1 (more than once a day) and Group 3 (once a week), *p* < 0.001, and between Group 1 (more than once a day and Group 4 (less than once a week), *p* < 0.001.

### 3.3. Sociodemographic Factors Affecting the Frequency of Internet Use

Multiple linear regression was carried out to determine the sociodemographic factors affecting the frequency of internet use in the sample. Assumptions regarding normality were met. Multicollinearity, based on the variance inflation factor, was acceptable. The regression model was significant *F*(9, 3479) = 100.90, *p* < 0.001. The adjusted R^2^ indicated that 21% of the variance in the frequency of internet use was explained by the model. Living alone/not alone, age, EIMD 2015 score, current employment situation, highest education level, and wealth quintile were all significant predictors of frequency of internet use (Table 2). More frequent internet use was reported amongst people who were not living alone, and individuals who were employed had higher education levels, higher wealth quintile, and higher EIMD 2015 scores. Regarding the age effect, increasing age was associated with less frequent use.

### 3.4. Type of Internet Use and Subjective Loneliness

Multiple linear regression was carried out to determine the effect of the type of internet use on subjective loneliness amongst the participants who reported using the internet more than once a month. All types of internet use were entered as predictors, alongside all the sociodemographic variables. Assumptions regarding normality were met, and multicollinearity was acceptable. The regression model was significant *F*(18, 3099) = 28.06, *p* < 0.001. The adjusted R^2^ indicated that 14% of the variance in subjective loneliness could be explained by the model. The analysis indicated that using the internet for e-mail communication and for searching for health-related information were significant predictors in the model, but none of the other types of use was significant (Table 3). E-mail use was associated with lower loneliness while using the internet to search for health-related information was associated with increased subjective loneliness. Amongst the sociodemographic predictors, not living alone, being male, being employed, and having higher EIMD 2015 scores and wealth quintiles were all associated with lower subjective loneliness.

Video or voice calls = Making video or voice calls; Health-r. information = Finding information about health-related issues; Shopping = Shopping/buying goods or services; Social networking = Using social networking sites; Reading news = Reading news, newspapers, and blogs; Streaming = Streaming TV, videos, and radio; Government info = Getting information about government services.

### 3.5. Gender Effects on the Type of Internet Use

We conducted separate ANOVAs to look at the effects of gender on the type of internet use. We found that there was more e-mail use by males than by females *F*(1, 4200) = 4.80, *p* = 0.03. This same pattern could be found in streaming, *F*(1, 4200) = 6.68, *p* = 0.01. Additionally, males read more news than females did, *F*(1, 4200) = 25.95, *p* < 0.001, and men searched more about government services than women, *F*(1, 4200) = 6.63, *p* = 0.01. Men were also using the internet more than women for managing finances, *F*(1, 4200) = 36.39, *p* < 0.001 (Table 4 includes a summary of means and standard errors). However, females searched more than males for health-related information, *F*(1, 4200) = 28.60, *p* < 0.001, and they used internet for social networking more than men, *F*(1, 4200) = 32.74, *p* < 0.001. Furthermore, women used the internet for video/voice calls more than men did, *F*(1, 4200) = 23.49, *p* < 0.001. Finally, there were no gender differences in shopping for goods or services (Table 4).

## 4. Discussion

The current study investigated the relationship between internet use (both frequency and purpose of use) and loneliness in a sizeable UK sample of adults aged 55–75, based on self-reported data from the ELSA COVID-19 sub-study (June/July 2020). We also further explored the sociodemographic factors which affected the frequency of internet use within this sample and explored gender differences regarding the type of internet use reported. In relation to frequency and purpose of internet use, we predicted associations between more frequent internet use and lower levels of loneliness as per previous (pre-pandemic) findings [9,16,17]. Additionally, we hypothesized that using the internet for e-mail communication would be associated with lower levels of loneliness, consistent with earlier findings where internet use for communication purposes was associated with a higher quality of life scores [22]. Furthermore, we predicted that using the internet to search for health-related information would associate with increased loneliness since previous work [21,22] has identified negative associations with life satisfaction.

We found a clear relationship between the frequency of internet use and loneliness during the pandemic. Those who used the internet more than once a day reported being less lonely than those who used the internet once a week or less. Previous cross-sectional findings suggest that increasing older people’s internet use could be beneficial for reducing loneliness, which could, in turn, lead to a range of positive effects on health [16,17,20]. This is especially paramount in the context of the COVID-19 restrictions which have significantly increased older people’s loneliness and isolation, with a substantial impact on mental health [29]. Despite restrictions now starting to ease in many countries, fostering internet use amongst older adults could be useful for addressing their detrimental long-term consequences, as well as isolation amongst older age groups more generally, which was an area of major concern even pre-pandemic [8,30].

As part of our investigation, we also looked at the risk factors underlying the digital divide in the sample. Higher age was associated with less frequent internet use; significantly less frequent use was also seen amongst individuals who lived alone, people who were not employed, had lower education levels, had lower wealth quintile, and lower EIMD 2015 scores. These factors are in line with earlier pre-pandemic findings [14,31]. Accessing digital services is essential for engaging and interacting with contemporary society [32], and during the pandemic, people have become even more reliant on the internet to access services and health information and to stay connected with friends and family. Consequently, those who were digitally excluded faced even greater access issues for medical appointments, support services, and consumer activities, further exacerbating the negative health impact of the pandemic, increasing isolation and loneliness, and social exclusion. While internet usage amongst the UK population, in general, surged to record levels during the pandemic [33], our findings show that the factors underpinning the digital divide remain broadly the same within the age range under study here: older people, those who lived alone, and individuals in lower socioeconomic groups, were less likely to use the internet [34]. Freedman and Nicolle (2020) highlighted the importance of primary care providers in identifying the individuals who are at risk of loneliness and offering effective interventions [6].

When looking at the impact of different types of internet use on loneliness, those who used the internet for e-mail communication purposes reported feeling less lonely. Previous findings have suggested that greater use of the internet for communication purposes is associated with lower levels of social loneliness [35]. However, the Sum et al. (2008) study only included five broad aspects of internet use (finding new people, entertainment, commerce, communication, and seeking information) unlike the current study where nine specific use types were considered; the present data differentiated between e-mail communication and video and voice calls, for example. Using the internet for e-mail communication seemed to help individuals to compensate for the strict pandemic-related social restrictions, allowing people to feel less lonely. Regarding gender differences in usage patterns, we found that males used the internet for e-mail communication more than females did. However, internet use for voice or video calls had no significant effect on loneliness. Hall et al. (2021) have found that voice calls are associated with less stress and loneliness. Video calls, on the other hand, can exacerbate loneliness because it might be emotionally challenging to see a missed loved one on a screen, as shown by a study into how different communication modalities affect the ability to cope with the social restrictions during the pandemic [36]. The current study conflated voice and video calls as part of the same internet use category, and this is perhaps why we could not find statistically significant effects: voice and video calls might have had opposing effects on loneliness levels and future studies would be advised to take this into account when designing their survey measures.

Social restrictions during the COVID pandemic have led to an increase in reliance on online social media channels, influencing mental health and well-being [37]. Importantly, the current study included social networking use as a separate use category, but we did not find any associations between older people’s use of social media and loneliness. There are mixed empirical findings in this regard. According to Geirdal et al. (2021), more frequent social media use during the pandemic was associated with significantly poorer mental and psychosocial health, increased loneliness, poorer well-being, and lower quality of life for adults [38]. On the other hand, Zhang et al. (2021) found that frequent social media use amongst older people was associated with lower levels of loneliness; this relationship was mediated by social contact and perceived social support [39]. Increased use of social media has been linked to an expansion of social networks, additional connections with peers [40], and access to a supportive online community [41]. Thus, it is possible that the positives and the negatives of social media usage combined have led to the net effect of no significant benefit for loneliness seen here. Likewise, Bell et al. (2013) found no significant difference in loneliness levels between older people who used social media versus those who did not [42]. Further work is needed to investigate which aspects and types of social media use might be beneficial for tackling isolation amongst older people to guide recommendations.

When the internet was used for information searches about health, participants reported higher levels of loneliness as per previous positive associations found pre-pandemic [21]. Additionally, the current study found that females used the internet to search for health-related information more than males did, confirming pre-pandemic findings [27]. A recent study found that internet use for searching for health-related information has been associated with higher depression levels during the pandemic [22]. Whilst depression and loneliness are separate constructs, loneliness is identified as a key risk factor for depression [43], and, as such, it does not come as a surprise that health-related information searching on the internet is associated with increased loneliness.

The present study provides valuable insight by exploring the effects of internet use on loneliness in a large sample of middle-aged and older adults. The study addressed a current lack of investigations focusing on the effects of internet use and loneliness amongst older people during the pandemic. Importantly, the dataset contained information regarding the *type* of internet use, which allowed us to draw insights based on this.

## 5. Limitations

Whilst the current study provides valuable detail regarding the relationships between internet usage, loneliness, and sociodemographic factors under lockdown conditions in older people, it does have limitations. For subjective loneliness, participants were asked one question only (‘How often do you feel lonely?’). A more comprehensive measure (e.g., the UCLA 3-item measure of loneliness [44]) would have been preferable. Additionally, the study relied on self-report data regarding internet usage and used a cross-sectional design. Studying the relationships being interrogated here using a longitudinal design (and, if possible, more objective measures) would benefit the field and allow yet more powerful inferences to be drawn.

## 6. Conclusions

To conclude, the current study found highly significant relationships between internet use frequency and loneliness when COVID-related social restrictions were in place amongst middle-aged and older UK adults. Although we cannot infer causality from the current study, pre-pandemic longitudinal research has shown that interventions promoting internet use amongst older adults can reduce depression and loneliness [45]. Here, the internet for e-mail communication purposes was associated with lower loneliness levels, while internet use for searching for health-related information had the opposite effect. No effects were found for other usage, such as social networking. The results of the current study support the idea that promoting more frequent internet use, particularly for e-mail use, could help counter loneliness amongst middle-aged and older people, particularly if COVID-related social restrictions continue to have to be reimposed in the future. However, the results regarding health-related information searching also highlight the potential downsides and negative effects of internet use. Health-related information searches could exacerbate anxiety and worry [46], leading to increased loneliness. Data regarding gender differences in the type of use are lacking in the literature, and the current study addressed this knowledge gap. We found that males report using the internet for e-mail more than females, while females’ use for health-related information searches was higher than in males. The current study also provides valuable information about the sociodemographic factors underlying the digital divide in older people, helping to identify groups at the highest risk of being digitally excluded. Overall, the current study findings are important for informing any intervention plans and policies (e.g., embedding digital inclusion into care planning, understanding the importance of group sessions, and the use of peer mentors) that aim to promote well-being and reduce loneliness by facilitating internet access amongst middle-aged and older people [47].

## Figures and Tables

**Table 1:** Summary of Participant Characteristics (*n* = 3500).

Measures	*M* (*SD*) or %	
Age (years)	67.20 (5.54)	
Gender	Male 43%	Female 57%
Living Alone/Not alone	Alone 22%	Not alone 78%
Urban/Rural living	Urban 73%	Rural 27%
Ethnicity	BAME 4%	Non-BAME 96%
Wealth quintile (1–5)	3.37 (1.39)	
EIMD 2015 score (1–5)	3.39 (1.32)	
**Current employment:**		
(1) Employed	25%	
(2) Unemployed	4%	
(3) Retired	65%	
(4) Other	6%	
**Highest education:**		
(1) Degree level	25%	
(2) Higher education	17%	
(3) Secondary school	33%	
(4) Below secondary school	25%	

*Note.* EIMD 2015 score = The Index of Multiple Deprivation 2015 is the official measure of relative deprivation for small areas and neighborhoods in England 1 (most deprived)–5; Wealth quintile = net financial wealth 1 (least affluent)–5. BAME = Black, Asian, and minority ethnic.

**Table 2:** Regression Model with Frequency of Internet Use as the Criterion Variable.

Measures	Unstandardized Coefficients	Standardized Coefficients	*t*	*p*
Constant	−0.98		−2.62	0.01
Living alone	−0.24	−0.07	−4.29	0.001
Age	0.05	0.17	10.44	0.001
Gender	−0.02	−0.01	−0.37	0.71
EIMD 2015 score (1–5)	−0.14	−0.12	−7.38	0.001
Urban/Rural living	−0.03	−0.01	−0.49	0.62
Employment	0.03	0.04	2.30	0.02
Highest education	0.23	0.31	19.13	0.001
Ethnicity	0.21	0.03	1.70	0.09
Wealth quintile (1–5)	−0.11	−0.10	−6.03	0.001

*Note.* Living alone = living alone or not alone; EIMD 2015 score = The Index of Multiple Deprivation 2015 is the official measure of relative deprivation for small areas and neighborhoods in England 1 (most deprived)–5; Highest education 1 = highest, Ethnicity = Non-BAME or BAME; Wealth quintile = net financial wealth 1 (least affluent)–5.

**Table 3:** Regression Model with Subjective Loneliness as the Criterion Variable.

Measures	Unstandardized Coefficients	Standardized Coefficients	*t*	*p*
Constant	1.90		11.41	0.001
Living alone	−0.38	−0.27	−15.47	0.001
Age	−0.001	−0.01	−0.51	0.61
Gender	0.14	0.12	6.75	0.001
EIMD 2015 score	−0.02	−0.04	−2.25	0.03
Urban/Rural living	−0.03	−0.03	−1.46	0.15
Employment	0.03	0.11	5.77	0.001
Highest education	0.01	0.03	1.77	0.08
Ethnicity	0.10	0.03	1.86	0.06
Wealth quintile	−0.02	−0.05	−2.71	0.007
E-mail use	−0.07	−0.04	−2.10	0.04
Video/voice calls	−0.02	−0.02	−1.09	0.28
Health-r. information	0.10	0.09	4.88	0.001
Managing finances	−0.01	−0.01	−0.39	0.70
Shopping	−0.01	−0.01	−0.44	0.66
Social networking	−0.01	−0.01	−0.34	0.73
Reading news	−0.03	−0.02	−1.21	0.23
Streaming	0.01	0.01	0.52	0.60
Government info	0.02	0.02	0.84	0.40

*Note.* Living alone = living alone or not alone; EIMD 2015 score = The Index of Multiple Deprivation 2015 is the official measure of relative deprivation for small areas and neighborhoods in England 1 (most deprived)–5; Highest education (1 = highest), Ethnicity = Non-BAME or BAME; Wealth quintile = net financial wealth 1 (least affluent)–5.

**Table 4:** Means and Standard Errors (Main Effect of Gender on the Type of Internet Use).

Measures	Males		Females		*p*
	*M*	*SE*	*M*	*SE*	
E-mail use	0.91	0.01	0.88	0.01	0.03
Video/voice calls	0.62	0.01	0.69	0.01	0.001
Health-r. information	0.41	0.01	0.49	0.01	0.001
Managing finances	0.68	0.01	0.59	0.01	0.001
Shopping	0.79	0.01	0.76	0.01	0.06
Social networking	0.48	0.01	0.56	0.01	0.001
Reading news	0.64	0.01	0.56	0.01	0.001
Streaming	0.53	0.01	0.49	0.01	0.01
Government info	0.43	0.01	0.40	0.01	0.01

*Note.* Video/voice calls = Making video or voice calls; Health-r. information = Finding information about health-related issues; Shopping = Shopping/buying goods or services; Social networking = Using social networking sites; Reading news = Reading news, newspapers, and blogs; Streaming = Streaming TV, videos, and radio; Government info = Getting information about government services.

## Data Availability

Data are publicly available at https://beta.ukdataservice.ac.uk/datacatalogue/series/series?id=200011. The data were accessed under project number 206540 on 1 January 2021.
